# Dietitians' utilization, attitudes, and experiences towards low-energy diets and very low-energy diets in dietary treatment of obesity

**DOI:** 10.1038/s41366-025-01780-y

**Published:** 2025-04-22

**Authors:** Caroline Bruun Abild, Anne-Louise Karstoft Klein, Trine Klindt, Jens Meldgaard Bruun, Dorthe Dalstrup Pauls

**Affiliations:** 1 Steno Diabetes Center Aarhus, Aarhus University Hospital, Aarhus, Denmark; 2 Department of Clinical Medicine, Aarhus University, Aarhus, Denmark; 3 Danish National Center for Obesity, Aarhus, Denmark; 4 Department of Health, Midwifery Program, University College South Denmark, Esbjerg, Denmark; 5 Danish Association of Clinical Dietitians, Copenhagen, Denmark

**Keywords:** Nutrition, Public health, Weight management, Nutrition therapy

## Abstract

The prevalence of overweight and obesity is increasing, and effective weight management care is needed. The present cross-sectional study aims to investigate the utilization, attitudes, and experiences of Low-Energy Diets (LED) and Very Low-Energy Diets (VLED) in the treatment of severe obesity among Danish clinical dietitians. Additionally, it seeks to identify barriers and motivation to implement these diets, and evaluate the need for additional resources and training among dietitians. In total, 76 Danish dietitians were included. Only 16% of participants currently employ LED or VLED, a much lower rate compared to similar international contexts. The primary barriers identified include doubts about long-term effectiveness, a concern for inducing disordered eating, and a preference for gradual lifestyle changes as recommended by health authorities. Responses highlighted a demand for additional training to boost dietitians’ confidence and understanding of these dietary strategies. In conclusion, this study highlights a need for enhanced educational efforts and resources to better integrate LED and VLED into obesity treatment in Denmark. It recommends focusing on patient-centered and individualized treatment approaches to address concerns and improve dietitians’ practical experiences, with the potential to include these diets in the overall treatment of obesity in Denmark.

## Introduction

The World Obesity Federation predicts that 51% of the global population will be living with overweight or obesity by 2035, contributing with a total economic impact of US$ 4.32 trillion [1]. Bariatric surgery and pharmacotherapy are currently the most effective treatments for obesity [2–4], but low-energy diets (LED) and very-low-energy diets (VLED) contribute to significant initial weight loss, potentially enhancing motivation and facilitating engagement in physical activities among clients [5]. The utilization of LED/VLED in the treatment of obesity are, however, controversial as they contradict health authorities’ recommendations of small lifestyle changes towards a slow weight loss [6], and the emerging trend toward “health at every size” or weight-neutral health [7].

In Denmark, dietitians are recognized as the primary healthcare professionals managing dietary treatment based on clinical guidelines [8, 9]. A British study has shown that UK dietitians perceived LED/VLED as effective, but economic concerns and doubts about long-term effectiveness were significant barriers to its widespread implementation [10]. There is limited knowledge regarding dieticians’ usage, experiences and attitudes towards LED/VLED, which is essential for providing effective weight management care. Therefore, the present study aimed to explore the utilization, attitudes, and potential opportunities and barriers towards using LED/VLED among Danish dietitians.

## Methods

The questionnaire used was based on the aforementioned British study [10]. Participants were primarily asked categorical questions, and asked to rate their understanding, motivation, and confidence towards using LED/VLED on a scale from 1–10, with 10 representing the highest possible rating. Some questions were followed by an open-ended question, allowing participants to contribute with their own perspectives.

Dieticians were recruited through social-media platforms, newsletters, email, and at relevant conferences between September 2022 to March 2023. Participants who were not fully qualified dieticians or who did not both provide demographic information and complete the LED/VLED questionnaire were excluded.

The questionnaires were distributed through an online link. Data were stored in anonymized form, ensuring compliance with the General Data Protection Regulation.

### Statistics

Categorical data are presented as percentage (%). Non-normally distributed continuers data are reported as median and interquartile range [IQR].

The association between usage of LED/VLED, and demographic characteristics were analyzed using Chi^2^ statistics. A Wilcoxon Mann-Whitney test was performed to explore differences in attitudes towards using LED/VLED between users and non-users.

Data analysis was performed using STATA 18.5. A two-sided *p*-value below 0.05 was considered statistically significant.

Qualitative analysis was performed using an iterative and inductive reflexive thematic approach in accordance with Braun and Clark’s six steps [11]. Two researchers independently conducted a thematic analysis based on the responses to the open-ended questions [12]. Quotes from participants are presented as stated.

## Results

In total, 650 clinical dietitians were invited to participate, 97 entered the survey, and 553 (85%) clinical dietitians did not want to participate for unknown reasons. 21 were excluded due to missing or default data, leaving 76 participants (96% female) in the present study (see flowchart in Supplementary S1).

In total, 54% were employed at hospitals. On average, participants obtained their education 12.5 years ago [IQR 4;21.5 years]. Participants were working within various areas, with overweight/obesity (49%), diabetes (55%), underweight including eating disorders (37%), elderly (33%), and other unknown areas (43%) reported as the most common.

### Utilization, attitudes, opportunities, and barriers towards using LED/VLED

Only 16% of participants reported using LED/VLED in their current practice, and those using LED/VLED primarily worked with overweight (58%), obesity (83%), and bariatric surgery (25%). Participants reported using LED/VLED less than once a week (67%) or 1 to 2 times per week (33%) (Table 1).

**Table 1:** Utilization of low-energy diets (LED) and very low-energy diets (VLED).

Usage of LED/VLED (n:76)		
	Yes	16%
	No	84%
How often do dieticians use LED/VLED (n:12)		
	Less than once weekly	67%
	1-2 times weekly	33%
	3-4 times weekly	0%
	Every day	0%
Patient groups where dieticians use LED/VLED (n:12)		
	Overweight	58%
	Obesity	83%
	Type 2 diabetes	8%
	Bariatric surgery	25%
	Polycystic Ovaries Syndrome	0%
	Other	8%
Who should start LED/VLED treatment (n:76) (multiple answers possible)		
	Dieticians	84%
	Medical doctors	36%
	Nurses	3%
	Interdisciplinary team	47%
	Suppliers of products	1%
	All of the above	1%
	Other	0%
	Don’t know	7%
Most suitable counseling when starting LED/VLED treatment (n:76) (multiple answers possible)		
	Individual	55%
	Group	7%
	Combination of individual and group	45%
	Other	1%
	Don’t know	13%
Can LED/VLED treatment result in long-term weight loss (n:76)		
	Yes	37%
	No	45%
	Don’t know	18%
Barriers using LED/VLED (n:76) (multiple answers possible)		
	Price	16%
	Side effects / safety	23%
	Weight gain	16%
	Knowledge/education	18%
	Risk of developing eating disorder	34%
	Weight maintenance	68%
	Don’t know	18%
Who should pay for LED/VLED products (n:76)		
	State	5%
	Patients	36%
	Hospitals	1%
	Patient and place of treatment	26%
	Other	7%
	Don’t know	25%

84% reported dietitians as the most appropriate health care professionals to introduce clients to LED/VLED, and 55% considered individual counseling as the most suitable setting. Weight maintenance was perceived as the primary barrier to initiate LED/VLED. Only 37% of participants reported that LED/VLED has the potential to facilitate sustained weight loss, and the cost of LED/VLED products should be paid by clients themselves (36%) or partially by the client and the treatment facility (26%) (Table 1).

**Fig. 1:**
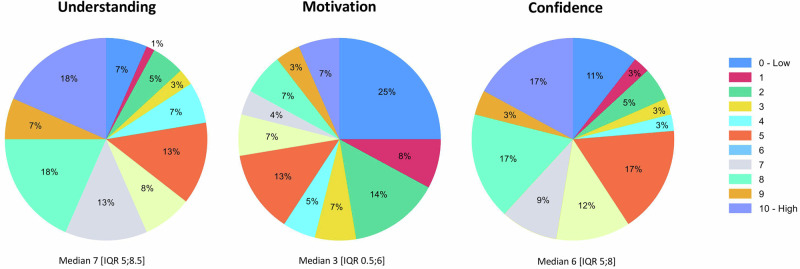
Understanding, motivation and confidence in using Low-energy diets/very low-energy diets in dietary treatment.

The understanding, motivation, and confidence in using LED/VLED in dietary treatment are illustrated in Fig. 1. On average, participants had a moderate to high understanding and confidence in using LED/VLED, but a low motivation to implement LED/VLED in dietary treatment.

Participants using LED/VLED exhibited a heightened understanding (8.5 vs. 6.5, *p* < 0.01), motivation (8 vs. 2, *p* < 0.01), and confidence (9.5 vs. 6, *p* < 0.01) in the utilization of LED/VLED, compared to participants who reported not to use LED/VLED. Dieticians who finalized their education less than five years ago utilized LED/VLED to a lesser extent than those with more than five years of experience (*p* < 0.05).

### Qualitative perspectives

Open-ended responses regarding motivation, confidence, understanding, and barriers to utilize LED/VLED can be found in Supplementary S2.

### Evidence and experience

Participants acknowledged the evidence supporting LED/VLED for weight loss but emphasized that practical clinical experience is equally important: *“I have clear knowledge about calculating needs, but not much experience with various products and how it affects clients.”* Many expressed a need for more knowledge, particularly around physiological and psychological aspects, and suggested workshops or networking to fill these gaps. Though most were confident in their understanding of LED/VLED, motivation to recommend it was mixed, with some viewing it as *“just another diet in a series,”* and not aligned with a holistic health perspective.

Those with extensive experience felt more motivated: *“My motivation is high, because I know it works!”* However, even motivated participants reflected on the need for organizational support to ensure optimal conditions for LED/VLED, suggesting, for example, dietitian support through general practice or links to obesity specialists for continued client assistance. Another participant highlighted the issue of weight regain post-program: *“There is a need for ‘rehab’ regarding weight maintenance… maybe attach dietitians via general practice.”*

### Concerns and risks

Concerns centred round the need for long-term, interdisciplinary support and the psychological impact of LED/VLED. Participants described challenges in providing adequate resources for reintroducing regular foods: *“…insufficient time to reintroduce regular foods after potential VLED interventions.”* Many were worried of the long-term effectiveness of LED/VLED: *“It makes no sense in most cases, especially when working with weight loss in municipalities, where it’s just a quick fix, and then the same work of eating normally comes afterwards.”*

Concerns about potentially fostering disordered eating also surfaced: *“It requires disordered eating behavior to maintain a significant weight loss in the long term.”*

### Individual assessment

Most participants agreed that LED/VLED decisions should be tailored to individual clients’ needs, motivation, and resources, stressing the importance of individualized treatment planning: *“It is individual and should be assessed with each client.”* Opinions varied on how to introduce LED/VLED; some felt clients should initiate the request and show motivation, while others considered health conditions like comorbidities in their decision-making. One participant highlighted this client-driven approach *“Only if there is motivation from the client, and rapid weight loss is desired/necessary.”*

## Discussion

Based on the present study, only a small proportion of Danish dietitians currently incorporate LED/VLED in their practice. However, a substantial proportion of the participants work within areas other than overweight/obesity in which utilization of LED/VLED cannot be expected. Concerns about the utilization of LED/VLED as a temporary ‘quick fix’ solution is raised by some participants. However, data from the DiRECT study show that a supervised VLED leads to sustained weight loss of over 6 kilograms and a 13% diabetes remission rate after 5 years [13].

In the present study, the proportion of participants utilizing LED/VLED is small, thus these results must be interpreted with caution. Nevertheless, participants emphasize the importance of evidence and practical experience, highlighting a need for ongoing education, potentially through workshops or collaboration in existing networks for dietitians underscoring the evidence grade for including meal replacements or liquid formula diets in weight loss treatment [8, 9]. Similar trends among dieticians in the field of obesity are reported in studies among Canadian and Australian dieticians [14, 15].

Some participants also emphasize the need to focus more holistically on the client’s well-being, and health, independent of weight. Recently, weight-neutral interventions have been developed as an alternative to the traditional weight-loss treatment. Current limited evidence suggests that weight-neutral programs are less effective to achieve weight loss, however, more effective to improve intuitive eating and reduce bulimic symptoms [7, 16].

In Denmark, health professionals, including dieticians, are recommended by the health authorities to assess a client’s needs through an interview during their initial meeting. This interview considers the client’s daily life, functional ability, health condition, risk factors, and motivation [17]. In line with this, participants underscore the need to tailor interventions based on the client’s motivation, needs, and resources. However, this study also suggests that LED/VLED is not being presented as a standard treatment option. Thus, the initiative to incorporate LED/VLED in treatment may depend on the client’s initiative.

It is evident that some of the perceived barriers for utilizing LED/VLED extend beyond short-term weight loss, encompassing issues related to long-term sustainability. Among UK dieticians, adherence (weight maintenance) was reported as a key barrier (57.6%) in line with our findings. However, contradictory to our findings, cost (price) was rated as the most important barrier among UK dieticians (66.1%), and risk of eating disorders as the third most important [8]. In the present sample, participants reported the risk of developing an eating disorder as the second most important barrier for utilizing LED/VLED. However, in a recent meta-analysis on this topic, authors found a decline in binge eating symptoms from pre- to post measurements in all included studies. Likewise, no studies reported aggravation in disordered eating symptoms throughout the interventions [18] underlining the importance of supervised interventions as potentially counteractive in aggravation of eating disorder risk factors. Nevertheless, future weight loss programs should address eating disorder issues, and prevention programs should strive towards a dual focus on obesity and eating disorder prevention [19]. Implementation of a brief screening tool to identify symptoms of eating disorders may be helpful to deliver an effective and secure weight loss treatment with LED/VLED.

The present study has some limitations (1) a limited response rate and a small group of participants using LED/VLED, (2) not all questions allowed open-ended answers, which would have deepened our understanding of the utilization, attitudes, and experiences towards LED/VLED, and (3) due to its cross-sectional design, it only allows for the reporting of associations and trends at a single time point. Thus the results may not be generalizable to other populations and countries. However, this study provides valuable insights into why LED/VLED is underused among dieticians.

## Conclusion

Further education and collaborative efforts to address concerns, enhance practical experience, and integrate LED/VLED more effectively into the overall treatment of severe obesity is needed. Patient-centered care, individualized assessments, and a nuanced understanding of LED/VLED should be emphasized in future interventions and educational initiatives. Moving forward, semi-structured interviews with clinical dieticians would provide valuable insights into current practice, utilization, and barriers towards using LED/VLED.

## Supplementary information

Supplementary

## Data Availability

The data are available from the corresponding author upon reasonable request.

## References

[1] World Obesity Federation. World Obesity Atlas 2023. Avaliable from: https://data.worldobesity.org/publications/?cat=19. Accessed October 1st 2024.

[2] Mentias A, Desai MY, Aminian A, Patel KV, Keshvani N, Verma S, et al. Trends and outcomes associated with bariatric surgery and pharmacotherapies with weight loss effects among patients with heart failure and obesity. Circ Heart Fail. 2024;17:e010453. 38275114 10.1161/CIRCHEARTFAILURE.122.010453

[3] Courcoulas AP, Gallagher JW, Neiberg RH, Eagleton EB, DeLany JP, Lang W, et al. Bariatric surgery vs lifestyle intervention for diabetes treatment: 5-year outcomes from a randomized trial. J Clin Endocrinol Metab. 2020;105:866–76. 31917447 10.1210/clinem/dgaa006 PMC7032894

[4] Sandsdal RM, Juhl CR, Jensen SBK, Lundgren JR, Janus C, Blond MB, et al. Combination of exercise and GLP-1 receptor agonist treatment reduces severity of metabolic syndrome, abdominal obesity, and inflammation: a randomized controlled trial. Cardiovasc Diabetol. 2023;22:41. 36841762 10.1186/s12933-023-01765-z PMC9960425

[5] Leslie WS, Taylor R, Harris L, Lean M. EJ. Weight losses with low-energy formula diets in obese patients with and without type 2 diabetes: systematic review and meta-analysis. Int J Obes. 2017;41:997. 10.1038/ijo.2017.46 PMC5467239 28290463

[6] Sundhedsstyrelsen. Små skridt til vægttab der holder. 2019. Avaliable from https://www.sst.dk/da/udgivelser/2019/Smaa-skridt. Accessed October 1st 2024.

[7] Dugmore JA, Winten CG, Niven HE, Bauer J. Effects of weight-neutral approaches compared with traditional weight-loss approaches on behavioral, physical, and psychological health outcomes: a systematic review and meta-analysis. Nutr Rev. 2020;78:39–55. 31393568 10.1093/nutrit/nuz020

[8] Hassapidou M, Vlassopoulos A, Kalliostra M, Govers E, Mulrooney H, Ells L, et al. European association for the study of obesity position statement on medical nutrition therapy for the management of overweight and obesity in adults developed in collaboration with the European Federation of the Associations of Dietitians. Obes Facts. 2023;16:11–28. 10.1159/000528083. 36521448 10.1159/000528083 PMC9889729

[9] Diabetes and Nutrition Study Group (DNSG) of the European Association for the Study of Diabetes (EASD). Evidence-based European recommendations for the dietary management of diabetes. Diabetologia. 2023;66:965–85. 10.1007/s00125-023-05894-8. 10.1007/s00125-023-05894-8 37069434

[10] Brown A, Brosnahan N, Khazaei D, Wingrove J, Flint SW, Batterham RL. UK dietitians’ attitudes and experiences of formula very low- and low-energy diets in clinical practice. Clin Obes. 2022;12:e12509. 35068081 10.1111/cob.12509 PMC9286801

[11] Braun V, Clarke V. Using thematic analysis in psychology. Qual Res Psychol. 2006;3:77–101.

[12] Braun V, Clarke V. What can “thematic analysis” offer health and wellbeing researchers? Int J Qual Stud Health Well-Being. 2014;9:26152. 25326092 10.3402/qhw.v9.26152 PMC4201665

[13] Lean ME, Leslie WS, Barnes AC, Brosnahan N, Thom G, McCombie L, et al. 5-year follow-up of the randomised Diabetes Remission Clinical Trial (DiRECT) of continued support for weight loss maintenance in the UK: an extension study. Lancet Diabetes Endocrinol. 2024;12:233–46. 38423026 10.1016/S2213-8587(23)00385-6

[14] Campbell K, Crawford D. Management of obesity: attitudes and practices of Australian dietitians. Int J Obes. 2000;24:701–10. 10.1038/sj.ijo.0801226 10878676

[15] Barr SI, Yarker KV, Levy-Milne R, Chapman GE. Canadian dietitians’ views and practices regarding obesity and weight management. J Hum Nutr Diet. 2004;17:503–12. 15546427 10.1111/j.1365-277X.2004.00562.x

[16] Mensinger JL, Calogero RM, Stranges S, Tylka TL. A weight-neutral versus weight-loss approach for health promotion in women with high BMI: A randomized-controlled trial. Appetite. 2016;105:364–74. 27289009 10.1016/j.appet.2016.06.006

[17] Sundhedsstyrrelsen. Anbefalinger for forebyggelsestilbud til borgere med kronisk sygdom. 2016. Available from: https://sundhedsstyrelsen.dk/da/nyheder/2016/~/media/23A6190B27B64822AB93B319903DDBB0.ashx. Accessed October 1st 2024.

[18] Jebeile H, Libesman S, Melville H, Low-Wah T, Dammery G, Seidler AL, et al. Eating disorder risk during behavioral weight management in adults with overweight or obesity: A systematic review with meta-analysis. Obes Rev. 2023;24:e13561. 36919475 10.1111/obr.13561 PMC10909435

[19] Stice E, Desjardins CD, Shaw H, Rohde P. Moderators of two dual eating disorder and obesity prevention programs. Behav Res Ther. 2019;118:77–86. 31005674 10.1016/j.brat.2019.04.002 PMC6540976

