# Supplementary material for: Dietitians' utilization, attitudes, and experiences towards low-energy diets and very low-energy diets in dietary treatment of obesity
Source: Int J Obes (Lond). 2025 Apr 22;49(7):1412–5. doi: 10.1038/s41366-025-01780-y (PMC12283391; doi:10.1038/s41366-025-01780-y)
Supplement: Supplementary file 1 — Supplementary [file 41366_2025_1780_MOESM1_ESM.docx]

**Supplementary**

**Supplementary S1:** Study flowchart

650 clinical dietitians were

invited to participate

97 participants entered the

survey

76 participants completed all

questionnaires

Participants excluded (n:21)

- Not a qualified dietician (n:1)

- Incomplete questionnaires (n:20)

553 clinical dietitians did not

want to participate

**Supplementary S2:** Qualitative (open-ended) answers

Motivation (n=29)

*How motivated are you to start a patient on VLCD/LCD?*

1. I do not work with that patient group.

2. I work with children, and those I see have other illnesses.

3. Limited confidence in the long-term effect and concerned about side effects.

4. It is too risky.

5. It is not my primary patient group.

6. It depends greatly on the individual. If the patient wants this, we start it, and if they do not, we focus on habit changes.

7. It must make sense and be relevant.

8. It works and provides good results. Many feel much better after starting.

9. Evidence of effect in the patient group.

10. I do not have the time to follow up on the course.

11. If I worked with the patient group, I would find it beneficial in some cases, but it would not be my first choice.

12. Lack of time to reintroduce normal food after a potential VLCD course.

13. I do not often suggest it unless the patient requests it.

14. I can suggest the option but never pressure people, as it is up to them which treatment they want to pursue.

15. I do not believe that a dietitian can responsibly offer a client/patient a diet with so many negative aspects.

16. I believe they will regain all the weight.

17. I would like more knowledge about the principles and safety of use in children before considering starting some patients.

18. I may be concerned that it only increases the patient's craving for other foods.

19. It depends on the circumstances and the person.

20. Only if the patient is motivated and rapid weight loss is desirable/necessary.

21. Only if it matches quality of life and adherence.

22. Unless there are contraindications such as illnesses, BED, etc.

23. Very little experience, not very familiar with the evidence.

24. When it is relevant.

25. Experience that it works for the patient.

26. Relevant for the relevant target group.

27. Need to be convinced that it is a healthy treatment in the long run as well.

28. It is easy, but not everyone has the resources. And it is "hard" for those who have a particular love for food.

29. My motivation is high because I know it works, but I experience that compliance is relatively low.

Confidence (n=19)

*How confident are you in your ability to start a patient on VLCD/LCD?*

1. It is up to the target group to choose what makes sense for them in their daily lives. VLCD can feel like a lonely path – especially if the person has a family that eats differently.

2. I believe I could do it, but it makes little sense in most cases where weight loss is addressed in municipalities—it is just a temporary fix, and then the same effort of eating normally follows.

3. It would be exciting and relevant to get an update, possibly a theme day on this.

4. This relates to my previous answer.

5. Physiological and psychological knowledge and experience.

6. If it became relevant, I would read up and seek information from trained dietitians in my network (e.g., through FAKD).

7. Again, I have no confidence in myself or other dietitians.

8. It is not something I have worked with much.

9. I am not good with such rigid plans.

10. I would never start a client on this.

11. Only if diabetes is involved. If it is just overweight, no.

12. I cannot assess the patient's biochemistry when working in a municipality, so I cannot detect potential deficiencies.

13. More knowledge needed.

14. Once I am familiar with the treatment, I see no problem.

15. Due to my extensive experience.

16. I need to read more about it.

17. It would require me to thoroughly review the literature.

18. When I assess it as the best approach, I always manage to motivate the patient.

19. I believe I am good at assessing who will be able to follow through and who will not.

Understanding (n=22)

*How would you assess your understanding of the principles of VLCD/LCD?*

1. No clinical experience with it.

2. Have used the diet <5 times.

3. Have experience and have used VLCD as a kickstart for dietary changes in research contexts.

4. Have not used it myself but have read about it.

5. Have known the product for many years and have used it, and the principles have not changed.

6. Have previously worked with these products and recommended them to my patients.

7. Have guided patients on it for many years and wrote my bachelor’s thesis on it.

8. Have knowledge of calculating needs but not much experience with the various products or how they affect patients.

9. In collaboration with patients.

10. No time or opportunity to delve into the dietary principles.

11. I do not work with it.

12. I do not use it in practice.

13. Have worked very little with VLCD/LCD.

14. Wrote my BA on the topic, gaining comprehensive understanding of the method.

15. I should use it more in practice.

16. Only theoretical understanding. No practical experience.

17. It has been a long time since I worked with it.

18. Many years of experience with VLCD interventions in research projects and dietary interventions in weight loss treatment.

19. Theoretically, it is easy to understand—important to screen for the right target group and note that the patient has a great responsibility in it.

20. During my studies, I interned at a private practice using VLCD. I independently guided clients during initiation as well as reintroducing normal food.

21. I have worked with it in various research projects and had colleagues using it in research (e.g., psoriasis, knee osteoarthritis school, and gout). However, I do not fully understand what happens to the microbiota or FFM 100%.

22. I need a refresher on potential side effects.

Barriers (n=36)

*What are your perceived barriers to use VLCD?*

1. Potential side effects include lack of energy, making physical activity difficult.

2. Most people find it challenging to maintain for extended periods if used as the sole source of nutrition. It is easier when replacing only some meals.

3. Rapid weight regain is common.

4. The transition from a liquid diet to regular food is difficult, and it does not help patients develop skills to manage normal food intake and the emotions underlying obesity.

5. Promotes yo-yo dieting and weight fluctuations.

6. Best suited for short-term use to avoid episodes of overeating.

7. Not a sustainable solution—only patients who can maintain a long-term diet should start VLCD. Primarily beneficial for pre-dialysis patients.

8. Should be followed by education on lifelong healthy eating and physical activity to be effective.

9. Group support helps—many patients doubt they can stick to it at first, but results can motivate them. However, not all can afford it.

10. No one should start this diet, according to some practitioners.

11. Concerns about long-term health impacts.

12. Weight loss is likely, but maintaining results is uncertain without strong support and guidance in transitioning back to normal eating habits.

13. Limited personal experience with VLCD, and rarely used in practice.

14. Some believe no one should use VLCD.

15. Lack of a response option to indicate strong opposition to VLCD, leading to uncertainty in survey responses.

16. Highly restrictive eating patterns are not optimal—often only work short-term, and weight is typically regained.

17. The root causes of obesity need to be addressed rather than just focusing on weight loss.

18. Lack of evidence for some patient groups.

19. Questions about the effectiveness and sustainability of VLCD.

20. Many patients who choose VLCD or LCD regain weight or develop/exacerbate binge eating disorder (BED).

21. Difficult for patients to maintain new habits.

22. Risk of weight gain after stopping the diet.

23. Taste issues with meal replacements.

24. Highly restrictive nature of the diet.

25. Behavioral habits are the biggest challenge in weight loss.

26. Disagreement among healthcare professionals regarding its use.

27. VLCD and LCD may work as a weight-loss kickstart but should be replaced by habit changes after a maximum of two months, as long-term adherence is difficult, and there is a risk of malnutrition.

28. VLCD does not address the underlying causes of obesity, raising concerns about long-term weight loss success and the potential risk of developing eating disorders or regaining weight.

29. Follow-up support is crucial.

30. Limited use in clinical practice—more research projects focus on VLCD rather than clinical implementation.

31. Need for more knowledge, recommendations, and clear guidelines for appropriate patient selection.

32. Weight loss places stress on the body, activating mechanisms that make maintaining weight loss challenging.

33. Poor adherence—many struggle to sustain such a low-calorie intake, making long-term success difficult.

34. Not enough time to guide patients through the reintroduction of regular foods.

35. Resistance from doctors and healthcare staff—patients’ social circles and personal fears about regaining more weight after stopping VLCD.

36. Ongoing follow-up is essential for sustained success.

Final Open-Ended Responses “Your Opinion” (n=18)

1. Both patient contact and support for behavior changes.

2. It is a good tool, but I do not think it suits everyone.

3. It is individual and should be assessed with each client/patient.

4. It is known that hunger and taste fatigue can occur with meal replacements, so a planned weight loss can also be achieved with minor dietary and exercise adjustments.

5. People can achieve 20-45 kg weight loss by eating a balanced diet tailored to their energy needs at normal weight.

6. Research shows that maintaining a large weight loss long-term requires disordered eating behaviors, which is why I do not support it.

7. In combination with patient contact and possibly maintaining 1-3 daily meal replacements.

8. In some cases, maintaining meal replacements.

9. My experience is mainly with patients using VLCD for rapid weight loss before bariatric surgery or as a 2-3 week start to boost motivation for weight loss through dietary changes and behavior modification.

10. I do not believe VLCD/LCD leads to lasting weight loss and that it unnecessarily "stresses" the body.

11. Most of my overweight clients need habit coaching and psychological support to find the root cause of overeating.

12. Exercise, social relationships, and close family play the biggest role in maintaining weight loss.

13. Frequent patient contact, realistic behavior changes, understanding how body and thoughts influence progress.

14. Psychological help would be beneficial.

15. Patients should participate in self-run support networks.

16. High risk of failure with VLCD; only a few people can lose weight and maintain it for life.

17. Important not just to treat symptoms.

18. We know patients regain weight after stopping. They need "rehab" for weight maintenance. Like medication, support should continue, even virtually.
